# The Writings of Hippocrates and Galen

**Published:** 1846-09

**Authors:** 


*The Writings of Hippocrates and Galen: Epitomised from the original Latin Translation.* By John Redman Cox, M. D., member of the Batavian Society of Sciences at Harlem; of the Royal Medical Society, and of the Royal Society of Sciences of Copenhagen, etc. Philadelphia, Lindsay & Blakiston, 1846; pp. 681.

We are indebted to the enterprising publishers, Messrs. Lindsay & Blakiston, for a copy of this truly interesting work. The medical profession especially, are certainly very greatly indebted to Dr. Cox, the learned translator and commentator, for it. As yet, however, we have hardly had time to more than glance over a few of its pages, and mere epitome as it is, of the writings of two of the greatest physicians that lived in ancient times, it will be read by every medical man, at least, who entertains a just love of his noble profession. It has long been to us a matter of surprise, that the writings of Hippocrates and Galen, which have been so much quoted and admired, have never been translated entire into the English language. An English copy of the works of these great men, voluminous as they are, would meet, we doubt not, with a ready and extensive sale.

"The gratification I have experienced," says the learned commentator, in "looking over the writings of these pioneers of medicine, has led me to believe that even this imperfect exhibition, may be acceptable to many, and that more especially, since few are likely to possess them in their complete and perfect form; yet it is necessary again to repeat, that to estimate the whole by this defective abstract, would be like one who judged of the character of a building by examining a brick which formed a fractional part of it."

To the dentist, having any love for general medicine, this work, which is brought out in a very neat and elegant style, will form a valuable acquisition to his library, and we advise all such, to lose no time in procuring a copy of it.

*Balt. Ed.*

